# Chronic Obstructive Pulmonary Disease is associated with risk of Chronic Kidney Disease: A Nationwide Case-Cohort Study

**DOI:** 10.1038/srep25855

**Published:** 2016-05-11

**Authors:** Chung-Yu Chen, Kuang-Ming Liao

**Affiliations:** 1 Department of Pharmacy, Kaohsiung Medical University Hospital, Kaohsiung, Taiwan; 2 School of Pharmacy, Master Program in Clinical Pharmacy, Kaohsiung Medical University, Kaohsiung, Taiwan; 3 Department of Internal Medicine, Chi Mei Medical Center, Chiali, Tainan City, Taiwan

## Abstract

Patients with chronic obstructive pulmonary disease (COPD) and chronic kidney disease (CKD) share common risk factors. However, there is limited information about COPD and CKD. This is case-cohort study was carried out using the Taiwanese National Health Insurance Research Database to evaluate the correlation between COPD and CKD. We identified cases aged older than 40 years who had an inpatient hospitalization with a first-time COPD diagnosis between 1998 and 2008. Control were selected from hospitalized patients without COPD or CKD and were matched according to age, gender, and year of admission at a 2:1 ratio. Cox proportional hazards model was used to assess the association of CKD and COPD. The overall incidence of CKD was higher in the COPD group (470.9 per 10^4^ person-years) than in the non-COPD group (287.52 per 10^4^ person-years). The adjusted hazard ratio of case was 1.61 (P < 0.0001) times that of control. COPD was found to be associated with kidney disease from our follow-up. To detect CKD early, early diagnosis of CKD in patients with COPD and prompt initiation of monitoring and treatment are imperative.

Chronic obstructive pulmonary disease (COPD) is a systemic inflammatory disease, and the airway obstruction is not fully reversible. Patients with COPD have a higher risk of comorbidities, including lung cancer, pulmonary tuberculosis, dementia, and coronary artery disease (CAD)^1^,^2^,^3^. These comorbidities may contribute to systemic inflammation in patients with COPD. Patients with COPD and chronic kidney disease (CKD) share common risk factors, including diabetes and hypertension^4^,^5^,^6^,^7^,^8^,^9^,^10^. Otherwise, patients with COPD are predisposed to have atherosclerotic disease via the inflammatory response, a condition that may also affect the renal vasculature, causing disease of small or large vessels and resulting in CKD^11^. However, the relationship between CKD and COPD has been largely undescribed. Only one study has shown the relationship between COPD and CKD in a cohort of vascular surgery patients with peripheral arterial disease^4^. There are no nationwide data in the literature concerning a relationship between COPD and CKD. Therefore, we aimed to clarify the incidence of CKD in patients with COPD and examine whether an association exists between COPD and CKD using the nationwide database in Taiwan.

## Results

### Baseline characteristics

**Figure 1:**
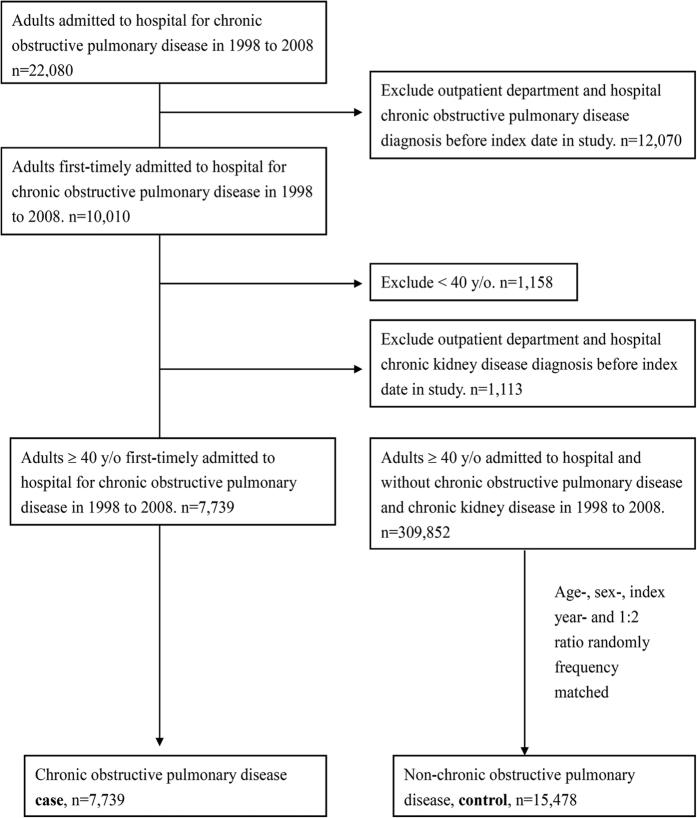
A flow chart that identifies the number of patients and study design.

The study population comprised 7,739 patients with COPD using hospitalization records from 1998 to 2008. Patient selection is illustrated in a flowchart (Fig. 1). The male predominant (67.48%) study population had a mean age and corresponding standard deviation (SD) of 71.73 (12.37) years, with >63% of the patients being above 70 years. In this cohort, stroke, CAD, hyperlipidemia, hypertension, diabetes, cancer, peripheral vascular disease (PVD), chronic liver disease, gout, sleep apnea were more prevalent in patients with COPD. Table 1 presents the comorbidities and demographic characteristics in the case and control group.

### Incidence of CKD

From Table 2, the incident rate of CKD per 10^4^ person-years and adjusted Hazard ratio (HR) between case and control are shown. The overall incident rate of CKD was higher in the case group than in the control group (470.9 vs. 287.52 per 10^4^ person-years). After adjusting for demographic characteristics and comorbidities, the adjusted HR of CKD in case was 1.61 (95% confidence interval (CI), 1.52–1.72; P < 0.0001) times that of the control group. This relationship was further investigated by examining the association between age and CKD. In both groups, the age-specific incidence of CKD raised with age. Comparing male patients in case group to control, the adjusted HR of CKD was 1.64 (95% CI, 1.54–1.80; P < 0.0001). Otherwise, female patients in the case group compared with the control group, adjusted HR of CKD was 1.54 (95% CI, 1.35–1.68; P < 0.0001).

### Subgroup analysis

**Figure 2:**
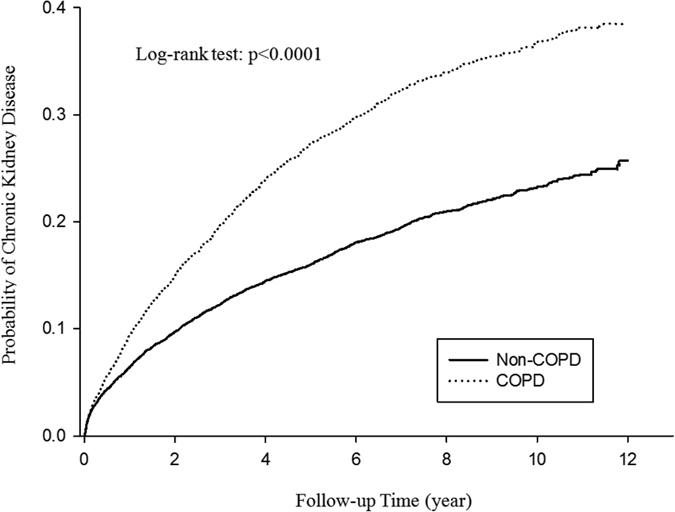
The cumulative incidence of CKD in patients with and without COPD.

In Table 3, the patients were stratified according to male at different age stratification, female at different age stratification, and with comorbidities at different age stratification. Among male patients with COPD, the adjusted HR of CKD increased with age, whereas among female patients with COPD, the adjusted HR of CKD was significantly higher in the patients aged 60–69 years (adjusted HR: 1.93; 95% CI, 1.52–2.44; P < 0.0001). Among patients with COPD and comorbidities, the adjusted HR of CKD increased with age. The cumulative incident rate of CKD in both cohorts is illustrated in Fig. 2. The incidence of CKD was significantly higher in the case group than in control (log-rank test, P < 0.0001).

## Discussion

To our knowledge, the present study is the first nationwide population-based study to address the association between COPD and CKD, and we provided the incidence rate of CKD in patients with COPD with a long-term follow-up period. In the study, patients with COPD have a 1.61 times higher risk of developing CKD than those without COPD, after adjusting for clinical risk factors. Previous studies^12^ have shown that baseline age, glomerular filtration rate (GFR), body mass index, diabetes, and smoking were related to the development of kidney disease. Otherwise, the long-term and averaged risk factors that were predictive of kidney disease included hypertension, hyperlipidemia, and diabetes. Our studies indicate that COPD is a risk factor for the development of CKD.

Only one study^4^ has reported that COPD is moderately associated with CKD in a population of vascular surgery patients, and moderate or severe COPD are related to increased mortality in patients with CKD. Our study case is a representative nationwide population of COPD compared with a matched cohort. In a previous study^4^, a borderline significant relationship was found between mild COPD and CKD, while moderate COPD had a higher risk of CKD (mild COPD and CKD: OR: 1.23, 95% CI, 0.99-1.53, P = 0.06; moderate COPD and CKD: 1.33, 95% CI, 1.07-1.65, P = 0.01) and no significant association was found between severe COPD and CKD.

In a previous study^13^, 300 patients with COPD were included, and their GFR was estimated. Patients were categorized according to their renal function as normal renal function, concealed chronic renal failure or overt chronic renal failure, and their prevalence rates were 54%, 26% and 20%, respectively. This was a single-center study with a small sample size, and the focus was on the chronic renal failure in patients with COPD. Another retrospective study^14^ was conducted between February 2006 and September 2007, and a total of 433 COPD patients and 233 subjects without COPD, were examined. The prevalence of having GFR < 60 was 9.6% in female patients with COPD and 5.1% in male patients with COPD patients. The authors reported that female sex, higher age, cachexia, and the inflammatory markers were associated with a higher risk of renal failure (GFR < 60) after multivariable analysis. The study also investigated the relationship between COPD and renal failure with a limited case number. Yoshizawa, *et al.*^15^ enrolled 108 patients with COPD and the prevalence of CKD was 31% with an odds ratio of 4.91. Our study showed the incidence of CKD in patients with COPD was 470.9 per 10,000 person-years and adjusted HR of CKD in patients with COPD was 1.61.

Smoking is an important risk factor for COPD^16^, and it also increases the risk of CKD^12^. Because smoking history was not available in our database, it is possible that some of the effects of CKD development observed in our study were due to the effect of smoking. In a cross-sectional, random-sampled interview survey, the female smoking rate was 4.8% in Taiwan^17^. In our study, the results of subgroup analysis included females only and revealed that COPD is still an independent risk factor of CKD with an adjusted HR 1.54 (95% CI, 1.35-1.68). Although the subgroup analysis cannot fully explain the confounding factor, the current findings still suggest that COPD itself or the systemic inflammation response plays an important role in the development of CKD.

Another finding in the present study is that the incidence of CKD increases with age in patients with COPD, regardless of gender and comorbidities. However, when stratified by gender and age, we found that female patients with COPD had the highest hazard of CKD in the population aged 60–69 years (adjusted HR: 1.93) compared with other age groups. A study including a nationally representative sample of noninstitutionalized adults^18^ showed that the prevalence of CKD rises dramatically with age. However, another study found that female patients with COPD have a higher mortality than males, suggesting that the protective effect of being female is lost in chronic obstructive pulmonary disease patients^19^. Together, these findings suggest all-cause mortality before the development of CKD in female patients with COPD older than 70 years and the highest incidence of CKD was found in the 60 to 69 years age group. The high incidence of CKD in the elderly might reflect a greater presence of other age-related CKD risk factors such as hypertension and diabetes in the general population and patients with COPD^20^. Otherwise, the development of CKD in the older age groups may be due to age-associated decline in renal function, which cannot be explained by other known risk factors^21^.

There are some possible reasons to delineate the relationship between COPD and CKD. First, patients with COPD may have coexistent diabetes or hypertension, possibly increasing the risk of CKD development. Previous studies have shown that patients with sleep apnea increased the likelihood of CKD whether meeting criteria for diabetes or hypertension^22^,^23^,^24^,^25^. After adjusting for important risk factors, patients with COPD have a higher risk of developing CKD in our study. Second, the inflammatory cytokine tumor necrosis factor-alpha^26^,^27^ was identified as a key mediator for atherosclerosis, and transforming growth factor (TGF)-β has been implicated in the development of COPD and atherosclerosis patients with COPD^28^,^29^,^30^. Atherosclerotic change was observed in the disease process of COPD. These cytokines are speculated to mediate the pathogenesis of COPD and CKD^4^. Although the finding has not yet been confirmed, it is possible that kidneys may be injured by damage to the blood vessels via the inflammatory process, increasing the risk of developing CKD. COPD are at increased risk of kidney injury especially among patients with hypoxemia and renal-endocrine mechanisms, tissue hypoxia, vascular rigidity have roles in the pathophysiology^31^.

The present study has some limitations. First, glomerular filtration rates, pulmonary function tests, and laboratory data were not available. Therefore, studies of the diagnostic accuracy of COPD and CKD will be criticized. Because there is no laboratory data at baseline, especially GFR or creatinine, there is a possibility that the baseline GFR of non-COPD is lower than that of COPD. Some of the limitations of the ICD codes as diagnostic criteria of CKD are that the severity of CKD are not clear, under diagnosis of CKD, the degree of smoking, dietary habits and the amount of nonsteroidal anti-inflammatory drugs are not known in the database. Because smoking history was not available in our database, it is possible that some of the effects of CKD development observed in our study were due to the effect of smoking and the portion of smoking in COPD might be higher than that in non-COPD, which might affect the results of this study. In the retrospective cohort study, selection bias and misclassification or information bias may occur. The ICD-9 codes used for COPD diagnosis have been extensively employed in other studies, and the internal findings also support the coding validity^32^,^33^,^34^. These criteria have been used in other studies and were previously described in detail^3^,^35^. Previous studies have used claims database to assess the epidemiology of chronic kidney disease^36^,^37^ and found that claims data can be used to identify patients with CKD because of a high positive predictive value^38^,^39^. Second, there is no staging of CKD and the severity of COPD. Because of the database limitation, we cannot explore staging of CKD following the diagnosis of CKD to facilitate appropriate treatment and monitoring of the patient. The association between COPD severity and CKD are also lacking. Third, non-COPD cohort seems to have a significantly different level of urbanization and urban residents may theoretically have a better access to health care and services than do residents in rural areas. This may reduce overall risk of developing CKD. We believe that the large number of samples drawn from the population and the exhaustive patient enrollment ensure that the data show a normal distribution and that the results are significant. Otherwise, we omission to include asthma as a relevant comorbidity. There has been increasing attention for the Asthma-COPD Overlap Syndrome (ACOS). Notably, a recent study showed that a concurrent asthma diagnosis was associated with lower risk of chronic kidney disease^40^.

Patients with COPD were found to be associated with kidney disease, and inflammation may contribute to the pathogenesis. Patients with COPD have a 1.6 times higher risk of developing CKD than those without COPD. The molecular mechanism of COPD and CKD needs more study to elucidate the pathway in more detail, and prospective studies with a longer follow-up duration are warranted to investigate the causality.

## Materials and Methods

### Data sources

In Taiwan, the National Health Insurance program was launched by a single-payer on March 1, 1995. Over 99.9% of Taiwan’s population were enrolled in the National Health Insurance program. This is case-cohort study was carried out using information from the National Health Insurance Research Database (NHIRD) of Taiwan, which contains encrypted computerized outpatient care claims, hospital inpatient care, ambulatory care, dental services, and prescription drugs records. Longitudinal Health Insurance Database (LHID) 2000 contains all original claim data of 1,000,000 individuals, all individuals randomly sampled from the 2000 Registry for Beneficiaries of the NHIRD, which continues the registration data of everyone who was a beneficiary of the National Health Insurance program during the period of 1996–2000^41^. This study was approved by the Institutional Review Board of Kaohsiung Medical University Hospital at Oct. 24, 2013 (KMUH-IRB-EXEMPT-20130010). Current NHIRD and hospital regulations and guidelines did not mandate informed consent in this retrospective cohort study. All procedures performed were in accordance with the ethical standards of the institutional research committee and with the directives of the Declaration of Helsinki.

### Study Designs

We identified patients aged older than 40 years who had an inpatient hospitalization with first-time COPD (ICD-9: 490–492, 496; A-code: A323 and A325) diagnosis between 1998 and 2008 from LHID 2000 as the case group. The date of the first-time COPD diagnosis was assigned as the index date.

We excluded patients who were diagnosed with COPD or CKD (ICD-9: 403–404, 580–587, 250.4, 274.1, 440.1, 442.1, 572.4 and 753.1) before the index date. The control group were selected from hospitalized patients without COPD or CKD and were matched according to age, gender, and the year of admission at a 2:1 ratio. Patients in this study were followed until a diagnosis of CKD was made, death occurred, December 31, 2009, was reached, or they withdrew from the national health insurance. Patient comorbidities were identified according to the diagnostic code as one inpatient diagnosis and one outpatient diagnosis one year before the index date (Supplementary eTable 1 online). Comorbidities included hypertension, diabetes, hyperlipidemia, chronic liver disease, stroke (ischemic and hemorrhagic), CAD, cancer, gout, PVD, and sleep apnea. Otherwise, we evaluated the demographic characteristics in patients with or without COPD, including gender, age stratification (40-49, 50-59, 60-69, and over 70 years), and urbanization.

### Statistics

The risk of CKD in the COPD and non-COPD groups was examined during the 11-year follow-up. Correlations with age, comorbidities and demographic characteristics were also determined in this study. All data were expressed as the frequency (percentage), mean and SD. Continuous and categorical variables were compared between the COPD and non-COPD groups using Student’s t-test and chi-square test, as appropriate. The incident rate of CKD was calculated as the number of CKD in total COPD patients and divided by the total person-years (per 10^4^ person-years). HR is a measure of relative risk over time in circumstances where we are interested not only in the total number of events, but in their timing as well. Therefore, we use cox proportional hazards model using univariate and multivariate analysis to assess the HR of CKD between the case and control groups during the follow-up. We also adjusted age group, gender, urbanization, hypertension, diabetes, hyperlipidemia, chronic liver disease, stroke (ischemic and hemorrhagic), CAD, cancer, gout, PVD, and sleep apnea in Cox proportional hazards model. To assess the robustness of the outcomes, a subgroup analysis was performed including age stratification (40–49, 50–59, 60–69, ≥70), gender (male/female), comorbidities (those with and without hypertension, diabetes, hyperlipidemia, chronic liver disease, stroke, CAD, cancer, gout, PVD, and sleep apnea), male at different age stratification, female at different age stratification, and with comorbidities at different age stratification. The difference in the cumulative incident rate of CKD between the case and control groups was calculated using Kaplan–Meier (KM) estimates. KM estimates were used to generate time-to-event curves for the each outcome and were tested using the log-rank test. All populations in which no predefined CKD or death occurred before the end of the study period (i.e., December 31, 2009) were censored. Analyses and calculations were performed using SAS ver. 9.4 (SAS Institute, Inc., Cary, NC, USA). Statistical significance was inferred at a two-sided p value less than 0.05.

## Additional Information

**How to cite this article**: Chen, C.-Y. and Liao, K.-M. Chronic Obstructive Pulmonary Disease is associated with risk of Chronic Kidney Disease: A Nationwide Case-Cohort Study. *Sci. Rep.* **6**, 25855; doi: 10.1038/srep25855 (2016).

## Supplementary Material

Supplementary Information

## Figures and Tables

**Table 1:** Demographic characteristics and comorbidities in patients with and without COPD.

Characteristics	Non-COPD (N = 15,478)	COPD (N = 7,739)	P-value
Age, years (mean ± SD)	71.72 ± 12.37	71.73 ± 12.37	0.9360
Age stratification (%)			
40–49	1026 (6.63)	1026 (6.63)	1.0000
50–59	1708 (11.04)	1708 (11.04)	
60–69	2974 (19.21)	2974 (19.21)	
≥70	9751 (63.12)	9751 (63.12)	
Gender (%)			
Female	5034 (32.52)	2517 (32.52)	1.000
Male	10444(67.48)	5222 (67.48)	
Urbanization*			
1 (highest)	4193 (27.09)	3062 (19.11)	<0.001
2	6560 (42.38)	1868 (43.79)	
3	3200 (20.67)	2409 (24.07)	
4 (lowest)	1525 (9.85)	400 (3.02)	
Comorbidities			
Stroke	2153 (13.91)	1542 (19.93)	<0.0001
Coronary artery disease	2629 (16.99)	1599 (20.66)	<0.0001
Hyperlipidemia	1823 (11.78)	782 (10.10)	0.0001
Hypertension	6350 (41.03)	3443 (44.49)	<0.0001
Diabetes	1777 (11.48)	795 (10.27)	0.0057
Cancer	1498 (9.68)	544 (7.03)	<0.0001
Gout	1226 (7.92)	581 (7.51)	0.2676
Peripheral vascular disease	255 (1.65)	163 (2.11)	0.0132
Chronic liver disease	1534 (9.91)	733 (9.47)	0.2877
Sleep apnea	723 (4.67)	504 (6.51)	<0.0001

^*^The level of urbanization was categorized into four levels based on the population density of the subject’s area of residence, where ‘1’ was most urbanized and ‘4’ was least urbanized.

**Table 2:** Incidence of CKD in patients with and without COPD, stratified by sex, age and comorbidity.

Characteristics	Non-COPD (N = 15,478)			COPD (N = 7,739)			Crude HR (95% C.I.)	p	Adjusted HR (95% C.I.)	p
	Event	PY	Incident Rate	Event	PY	Incident Rate				
**CKD**	2266	78810.90	287.52	1790	38012.62	470.90	**1.63 (1.53–1.73)**	**<0.001**	**1.61 (1.52–1.72)**	**<0.001**
Age stratification										
40–49	112	6361.27	176.07	83	3043.54	272.71	1.55 (1.17–2.06)	0.003	1.33 (0.99–1.79)	0.055
50–59	232	9434.92	245.90	174	4593.04	378.83	1.54 (1.27–1.88)	<0.001	1.43 (1.17–1.75)	<0.001
60–69	497	16604.79	299.31	385	8134.08	473.32	1.58 (1.38–1.81)	<0.001	1.57 (1.37–1.79)	<0.001
≥70	1425	46409.90	307.05	1148	22241.95	516.14	1.68 (1.56–1.82)	<0.001	1.65 (1.53–1.79)	<0.001
Gender										
Male	1502	51752.64	290.23	1212	25034.23	484.14	1.66 (1.54–1.79)	<0.001	1.64 (1.54–1.80)	<0.001
Female	764	27058.25	282.35	578	12978.38	445.36	1.55 (1.41–1.75)	<0.001	1.54 (1.35–1.68)	<0.001
Comorbidities										
Stroke										
Yes	362	9956.30	363.59	366	6725.91	544.16	1.49 (1.28–1.72)	<0.001	1.48 (1.28–1.72)	<0.001
No	1904	68854.59	276.52	1424	31286.70	455.15	1.64 (1.53–1.75)	<0.001	1.64 (1.53–1.75)	<0.001
Coronary artery disease										
Yes	455	12330.36	369.01	470	6966.46	674.66	1.83 (1.61–2.08)	<0.001	1.79 (1.57–2.04)	<0.001
No	1811	66480.53	272.41	1320	31046.15	425.17	1.59 (1.49–1.71)	<0.001	1.55 (1.44–1.67)	<0.001
Hyperlipidemia										
Yes	376	7985.86	470.83	251	3451.74	727.17	1.52 (1.30–1.79)	<0.001	1.54 (1.31–1.81)	<0.001
No	1890	70825.03	266.85	1539	34560.87	445.30	1.65 (1.54–1.76)	<0.001	1.62 (1.51–1.73)	<0.001
Hypertension										
Yes	1125	29363.41	383.13	952	15184.95	626.94	1.63 (1.49–1.77)	<0.001	1.64 (1.50–1.79)	<0.001
No	1141	49447.48	230.75	838	22827.66	367.10	1.58 (1.45–1.73)	<0.001	1.57 (1.43–1.71)	<0.001
Diabetes										
Yes	436	8537.46	510.69	292	3707.41	787.61	1.54 (1.33–1.79)	<0.001	1.47 (1.51–1.80)	<0.001
No	1830	70273.43	260.41	1498	34305.20	436.67	1.68 (1.57–1.80)	<0.001	1.62 (1.51–1.74)	<0.001
Cancer										
Yes	195	6881.16	283.38	116	2557.60	453.55	1.60 (1.27–2.01)	<0.001	1.63 (1.30–2.08)	<0.001
No	2071	71929.73	287.92	1674	35455.01	472.15	1.64 (1.54–1.75)	<0.001	1.61 (1.51–1.72)	<0.001
Gout										
Yes	263	5187.08	507.03	189	2415.83	782.34	1.55 (1.29–1.87)	<0.001	1.57 (1.30–1.90)	<0.001
No	2003	73623.81	272.06	1601	35596.79	449.76	1.64 (1.56–1.78)	<0.001	1.62 (1.51–1.73)	<0.001
Peripheral vascular disease										
Yes	49	1342.85	364.90	38	956.66	397.22	1.11 (0.73–1.68)	0.695	1.09 (0.70–1.69)	0.682
No	2217	77468.04	286.18	1752	37055.95	472.80	1.68 (1.58–1.79)	<0.001	1.62 (1.52–1.72)	<0.001
Chronic liver disease										
Yes	294	7722.66	380.70	213	3704.84	574.92	1.51 (1.27–1.80)	<0.001	1.46 (1.22–1.75)	<0.001
No	1972	71088.23	277.40	1577	34307.77	459.66	1.66 (1.55–1.77)	<0.001	1.62 (1.52–1.75)	<0.001
Sleep apnea										
Yes	115	2859.78	402.13	129	1945.00	663.24	1.84 (1.57–2.05)	<0.001	1.72 (1.54–2.01)	<0.001
No	2151	75951.12	283.21	1661	36067.62	460.52	1.64 (1.54–1.77)	<0.001	1.62 (1.52–1.75)	<0.001

PY, per 10,000 person-years; HR, hazard ratio.

**Table 3:** Incidence of CKD stratified by male or female at different age stratification, and with comorbidities at different age stratification, with the hazard ratio for patients with COPD compared with those without COPD.

Characteristics	Non-COPD (N = 15,478)			COPD (N = 7,739)			Crude HR (95% C.I.)	p	Adjusted HR (95% C.I.)	p
	Event	PY	Incident Rate	Event	PY	Incident Rate				
Male										
40–49	85	4009.75	211.98	52	1956.20	265.82	1.26 (0.90–1.78)	0.198	1.16 (0.81–1.66)	0.459
50–59	150	5838.01	256.94	113	2887.99	391.28	1.51 (1.18–1.93)	0.001	1.42 (1.11–1.83)	0.006
60–69	347	11036.78	314.40	243	5531.63	439.29	1.40 (1.19–1.65)	<0.001	1.43 (1.21–1.69)	<0.001
≥70	920	30868.10	298.04	804	14658.40	548.49	1.85 (1.66–2.01)	<0.001	1.81 (1.64–1.99)	<0.001
Female										
40–49	27	2351.52	114.82	31	1087.34	285.10	2.47 (1.47–4.15)	0.001	1.81 (1.03–3.16)	0.007
50–59	82	3596.91	227.97	61	1705.04	357.76	1.55 (1.11–2.12)	0.007	1.48 (1.05–2.09)	0.001
60–69	150	5568.01	269.40	142	2602.45	545.64	2.01 (1.60–2.54)	<0.001	1.93 (1.52–2.44)	<0.001
≥70	505	15541.80	324.93	344	7583.54	453.61	1.39 (1.21–1.59)	<0.001	1.39 (1.21–1.59)	<0.001
Comorbidities										
40–49	112	6361.27	176.07	83	3043.54	272.71	1.56 (1.18–2.04)	0.021	1.36 (1.02–1.81)	0.048
50–59	232	9434.92	245.90	174	4593.04	378.83	1.54 (1.27–1.88)	0.007	1.43 (1.17–1.75)	0.004
60–69	497	16604.79	299.31	385	8134.08	473.32	1.57 (1.37–1.80)	<0.001	1.58 (1.38–1.80)	<0.001
≥70	1425	46409.90	307.05	1148	22241.95	516.14	1.67 (1.55–1.83)	<0.001	1.61 (1.49–1.75)	<0.001

PY, per 10,000 person-years; HR, hazard ratio.
