# Supplementary material for: Chronic Obstructive Pulmonary Disease is associated with risk of Chronic Kidney Disease: A Nationwide Case-Cohort Study
Source: Sci Rep. 2016 May 11;6:25855. doi: 10.1038/srep25855 (PMC4863146; doi:10.1038/srep25855)
Supplement: Supplementary Information [file srep25855-s1.doc]

**Supplementary Materials**

**Chronic Obstructive Pulmonary Disease is associated with risk of Chronic Kidney Disease: A Nationwide Case-Cohort Study**

Chung-Yu Chen, Kuang-Ming Liao

eTable1. World Health Organization the International Classification of Diseases (ICD)-9-CM code numbers used for comorbidities

| Disease Category | ICD-9-CM Number |
| --- | --- |
| Hypertension | 401-405 |
| Diabetes | 2501,2502,2503,2504,2505,2506,2507,2508,2509 |
| Hyperlipidaemia | 272 |
| Chronic liver disease | 571 |
| Cancer | 140-208 |
| Stroke | 430,431,432, 435, 433,434,436,437,438, |
| Coronary artery disease (CAD) | 410-414 |
| Gout | 274 |
| Peripheral vascular disease (PVD) | 443, 44021 |
| Sleep apnea | 32720,32721,32722,32723,32724,32725,32726,32727,32728,32729,78051,78052,78053,78054,78055,78056,78057 |
